# A Blood Exosomal miRNA Signature in Acute Respiratory Distress Syndrome

**DOI:** 10.3389/fmolb.2021.640042

**Published:** 2021-07-15

**Authors:** Gilles Parzibut, Monique Henket, Catherine Moermans, Ingrid Struman, Edouard Louis, Michel Malaise, Renaud Louis, Benoît Misset, Makon-Sébastien Njock, Julien Guiot

**Affiliations:** ^1^ Department of Intensive Care, University Hospital of Liège, Liège, Belgium; ^2^ Laboratory of Pneumology, GIGA Research Center, University of Liège, University Hospital of Liège, Liège, Belgium; ^3^ Laboratory of Molecular Angiogenesis, GIGA Research Center, University of Liège, Liège, Belgium; ^4^ Laboratory of Gastroenterology, GIGA Research Center, University of Liège, University Hospital of Liège, Liège, Belgium; ^5^ Fibropole Research Group, University Hospital of Liège, Liège, Belgium; ^6^ Laboratory of Rheumatology, GIGA Research Center, University of Liège, University Hospital of Liège, Liège, Belgium

**Keywords:** acute respiratory distress syndrome, acute lung injury, diagnostic biomarkers, exosomes, microRNAs, inflammation, fibrosis

## Abstract

Acute respiratory distress syndrome (ARDS) is a diffuse, acute, inflammatory lung disease characterized by a severe respiratory failure. Recognizing and promptly treating ARDS is critical to combat the high mortality associated with the disease. Despite a significant progress in the treatment of ARDS, our ability to identify early patients and predict outcomes remains limited. The development of novel biomarkers is crucial. In this study, we profiled microRNA (miRNA) expression of plasma-derived exosomes in ARDS disease by small RNA sequencing. Sequencing of 8 ARDS patients and 10 healthy subjects (HSs) allowed to identify 12 differentially expressed exosomal miRNAs (adjusted *p* < 0.05). Pathway analysis of their predicted targets revealed enrichment in several biological processes in agreement with ARDS pathophysiology, such as inflammation, immune cell activation, and fibrosis. By quantitative RT-PCR, we validated the alteration of nine exosomal miRNAs in an independent cohort of 15 ARDS patients and 20 HSs, among which seven present high capability in discriminating ARDS patients from HSs (area under the curve > 0.8) (miR-130a-3p, miR-221-3p, miR-24-3p, miR-98-3p, Let-7d-3p, miR-1273a, and miR-193a-5p). These findings highlight exosomal miRNA dysregulation in the plasma of ARDS patients which provide promising diagnostic biomarkers and open new perspectives for the development of therapeutics.

## Introduction

Acute respiratory distress syndrome (ARDS) is a heterogeneous entity associating clinical, radiological, and pathological features leading to a severe decrease in lung diffusing capacity and compliance (Ashbaugh et al., 1967). ARDS is characterized by diffuse alveolar damage (DAD), associated with an increase in alveolar and capillary permeability resulting in an interstitial and alveolar edema that finally requires mechanical ventilation (Katzenstein et al., 1976; Mendez and Hubmayr, 2005; Cardinal-Fernández et al., 2017). The diagnosis of ARDS is based on the Berlin criteria (2012), stating that clinical presentation arises within seven days after exposure to a “common risk factor,” with pneumonia being the major cause (ARDS Definition Task Force et al., 2012). In up to 8% of ARDS cases, the leading cause of pathology is undetermined despite a systematic diagnosis approach, resulting in increased mortality rate. Thus, a methodical comprehensive work-up is necessary for ARDS of unknown origin.

The diagnosis is based on the association of DAD with deep hypoxemia. The prevalence is variable according to epidemiological studies but could reach up to 10.4% in patients admitted to the intensive care unit for a mortality of around 50% in the subgroup of patients suffering from severe ARDS (Bellani et al., 2016). In addition, survivors are at high risk of developing cognitive decline, depression, post-traumatic stress, and the classic side effects of long-term care, such as polyneuropathy, sarcopenia, or stroke disorder, sleep. In this context and in spite of significant progress in the field of mechanical ventilation and extra-corporeal respiratory assistance, it remains essential to identify early patients with ARDS in order to offer them the most appropriate therapy as soon as possible.

The lesion initially found in ARDS is the appearance of lesional edema resulting from an increased permeability to liquid and protein across the lung endothelium. Increased alveolar–capillary permeability results in accumulation of protein-rich fluid inside the alveoli, thereby producing diffuse alveolar damage (Bachofen and Weibel, 1982; Matthay et al., 2012). Then, the activation of resident alveolar macrophages will induce production of large amount of pro-inflammatory cytokines (interleukin (IL)-6, IL-8, tumor necrosis factor-a) at the origin of the recruitment of polynuclear neutrophils to the alveolar compartment and amplification of the inflammation response (Potey et al., 2019). Classically after 72 h, the repair processes are initiated, under the assumption of control of the etiopathogenic phenomenon at the origin of the lesion process. During this phase, there is intra-alveolar and capillary migration of (myo)fibroblasts and other progenitor cells with the aim of restoring the alveolar–capillary barrier ad integrum (Zemans et al., 2011; Dial et al., 2017). Once epithelial integrity is restored, the edema is resolved. The last phase is the recovery phase associating repair and scarring processes including fibroblastic activation. This fibrotic phase is particularly associated with a high morbidity–mortality rate due to its irreversibility and the association with an increased ventilation time. In this context, there is a high need for specific biomarkers helping clinicians to predict the evolution of patients (Thompson et al., 2017).

MicroRNAs (miRNAs) are small non-coding RNAs (19–22 nucleotides) which play a crucial role in the regulation of gene expression by repressing the translation of target genes or degrading their target messenger RNAs (mRNAs) (He and Hannon, 2004; Cheng et al., 2014; Njock et al., 2015; Guiot et al., 2020). miRNAs are secreted into body fluids (e.g., bronchoalveolar lavage fluid, saliva, sputum, and plasma) within extracellular vesicles, including exosomes (30–150 nm). The encapsulation of exosomal miRNAs into a bilayer lipid membrane protects them from degrading enzymes (e.g., ribonucleases) and confers them a remarkable stability in the bloodstream (Valadi et al., 2007; Li et al., 2015). The composition of exosomal miRNAs varies according to the state of their parental cells. In the pathological context, exosome content is robustly altered, including miRNAs, which represent a promising source of biomarkers (Guiot et al., 2019, 2020). Recently, Njock et al. (2019) identified a unique signature of three altered miRNAs (miR-142-3p, miR-33a-5p, and Let-7d-5p) from sputum of IPF patients. Several studies have reported an alteration of inflammatory-related exosomal miRNAs from airway biofluids (BALF and sputum supernatants) in the asthma context (Maes et al., 2016; Suzuki et al., 2016). In ARDS, only few studies have investigated the identification of altered circulating miRNAs, particularly exosomal miRNAs, and most of the works have focused on animal models, limiting knowledge on humans (Rahmel et al., 2018; Xu et al., 2020). Furthermore, there is no study on the characterization of exosomal miRNA content in ARDS. It is a systemic inflammatory disease, which suggests that blood exosomes generated by circulating cells (e.g., platelets and immune cells) as well as tissue cells (e.g., pulmonary, cardiovascular, renal, and hepatic) may participate in the pool of altered miRNA characteristics of ARDS.

In this study, we aimed to characterize small RNA content of plasma exosomes from ARDS patients in order to identify potential diagnostic biomarkers of the disease. For this, we profiled miRNA expression levels in plasma-derived exosomes from ARDS patients compared to healthy subjects (HSs) by small RNA sequencing (RNA-seq). This allowed us to identify nine differentially expressed (DE) miRNAs (miR-122-5p, Let-7d-3p, miR-24-3p, miR-130a-3p, miR-98-3p, miR-221-3p, miR-193a-5p, miR-1273a, and Let-7a-5p) in the ARDS context (in both screening and validation cohorts). The evaluation of their diagnostic potential showed that these DE miRNAs differentiate ARDS patients from HSs with a high level of accuracy. This study provides candidate biomarkers for the diagnosis of ARDS disease and opens new perspectives for the development of therapeutics.

## Materials and Methods

### Demographic and Clinical Characteristics of Discovery and Validation Cohorts

**FIGURE 1:**
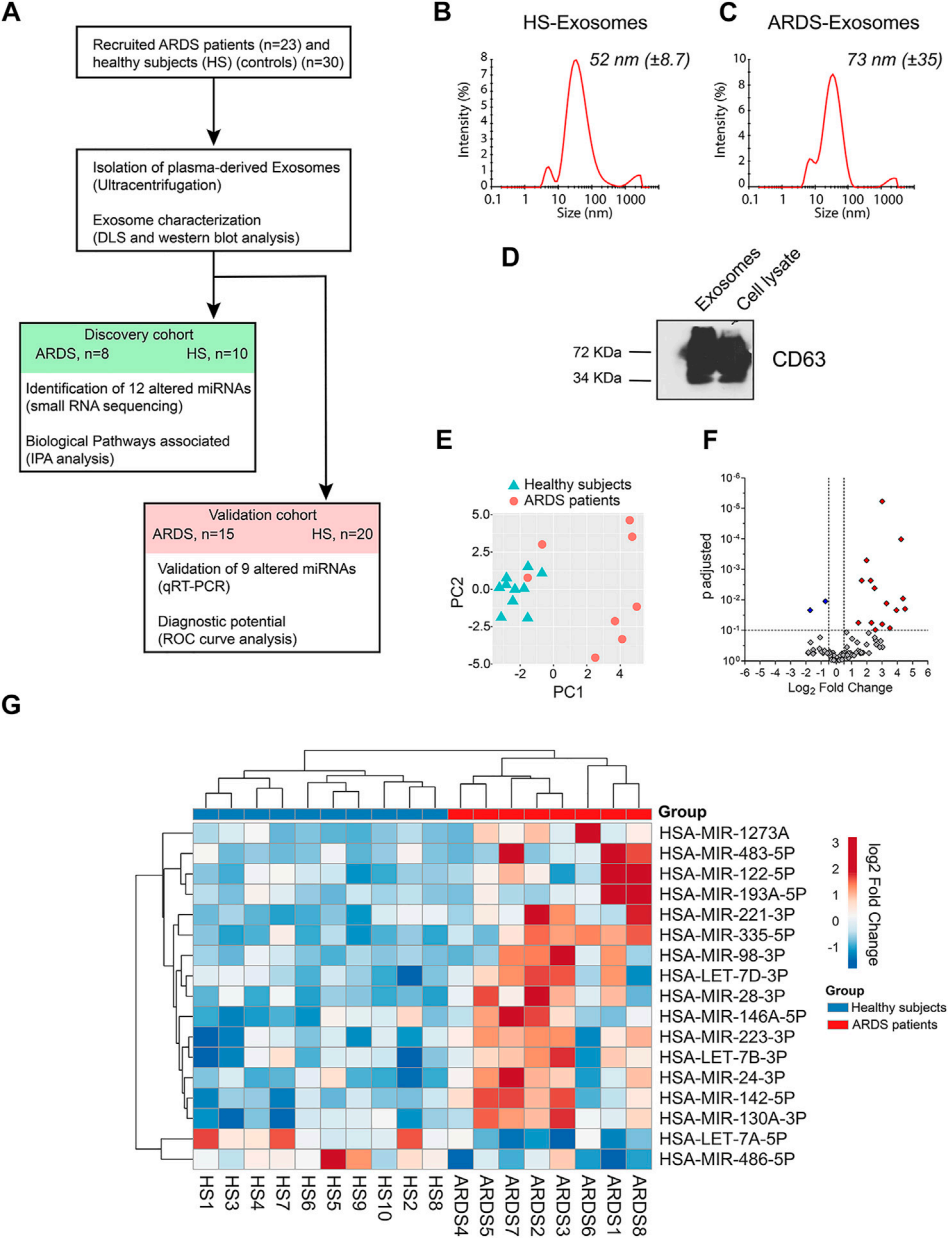
Identification of exosomal miRNAs altered in the plasma of ARDS patients. **(A)** Schematic representation of the approach used to identify the candidate miRNA biomarker for ARDS. ARDS: acute respiratory distress syndrome, DLS: dynamic light scattering, HS: healthy subjects, IPA: Ingenuity Pathway Analysis, and ROC: receiver-operating characteristic. **(B,C)** Representative size distribution of exosome-like vesicles isolated from the plasma of **(B)** HSs and **(C)** ARDS patients by dynamic light scattering (DLS) analysis. **(D)** Western blot analysis of the exosomal marker CD63 in lysates from plasma-derived exosomes and cell lysate. **(E)** Principal component analysis (PCA) plot on miRNA expression data showing separation of exosome samples from HS and ARDS plasma. **(F)** Volcano plot showing the differentially expressed exosomal miRNAs. 17 miRNAs present differential expression between HSs and ARDS patients (fold change > 1.4 and adjusted *p*-value < 0.1). 15 upregulated (red) and 2 downregulated (blue) miRNAs. **(G)** Heat map showing the expression of 17 altered miRNAs in 10 HSs (blue) and 8 ARDS patients (red) within the discovery cohort after unsupervised two-way hierarchical clustering.

Patients with a diagnosis of moderate to severe ARDS according to the definition of “Berlin” (ARDS Definition Task Force et al., 2012) were enrolled at the intensive care unit of University Hospital of Liège (Liège, Belgium). The protocol was approved by the Ethics Committee of CHU of Liège, and all subjects gave written consent before their enrollment (Belgian number: B707201422832, ref: 2014/302). The first cohort of 8 ARDS patients and 10 HSs (controls) was used to identify altered exosomal miRNAs in the ARDS context, and the second cohort of 15 ARDS patients and 20 HSs was used to validate miRNA candidates and study their diagnostic potentials (Figure 1A). Table 1 provides an overview of the demographic and clinical characteristics of the patients from the discovery and validation cohorts. ARDS patients and HSs from the two independent cohorts are age- and sex-matched.

**TABLE 1:** Clinical characteristics of the enrolled HSs and the ARDS patients.

	Discovery cohort		Validation cohort	
	HS (*n* = 10)	ARDS (*n* = 8)	HS (*n* = 20)	ARDS (*n* = 15)
Age (years)	55.2 ± 6.4	60.7 ± 11.4	62.6 ± 9.8	62.1 ± 11
Gender (M/F)	4/6	4/4	8/12	10/5
BMI	-	26.7 ± 4.3	-	27.7 ± 3.3
Weight (kg)	-	76.8 ± 14.7	-	80.8 ± 9.2
Height (cm)	-	168.8 ± 5.5	-	172.8 ± 5.5
In-hospital death by day 28	-	4/8 (50%)	-	6/15 (40%)
Total SOFA score	-	7.2 ± 2.5	-	9.2 ± 3.8
Vol Tidal (ml)	-	446.1 ± 56.1	-	410.1 ± 56.1
Tidal volume (ml/kg) of predicted body weight	-	6.8 ± 0.9	-	6.3 ± 0.4
FiO_2_	-	85 ± 12.9	-	75 ± 14
Driving pressure (cm H_2_O)	-	17.2 ± 3.1	-	14.9 ± 2.1
PEEP (cm H_2_O)	-	12.1 ± 1.8	-	12.2 ± 2.2
PaO_2_/FiO_2_	-	89.8 ± 31.6	-	130 ± 31.6
Prone position	-	8/8 (100%)	-	10/15 (66%)
Neuromuscular blockade	-	8/8 (100%)	-	10/15 (66%)
NO	-	0/8 (0%)	-	1/15 (13%)
ECMO V-V	-	1/8 (12.5%)	-	1/15 (6%)

Data are presented as mean ± SD or median (IQR). ARDS: acute respiratory distress syndrome, BMI: body mass index, F: female, ECMO V-V: extracorporeal membrane oxygenation (veno-venous), FiO_2_: fraction of inspired oxygen, HSs: healthy subjects, H_2_O: water, M: male, NO: nitric oxide, PaO_2_/FiO_2_: pressure of arterial oxygen to fractional inspired oxygen concentration, PEEP: positive end-expiratory pressure, and SOFA scores: Sequential Organ Failure Assessment scores measured in five organ systems (respiratory, cardiovascular, hematologic, gastrointestinal, and renal; the neurologic system was not assessed), with each organ score ranging from 0 to 4, resulting in an aggregated score that ranges from 0 to 20, with higher scores indicating greater dysfunction.

### Plasma Processing and Exosome Isolation

Blood was collected and transferred to EDTA-containing tubes. Plasma was isolated from the blood by centrifugation at 1,500 rpm for 10 min at 4°C to remove blood cells, and the supernatant was centrifuged at 2,000 rpm for 15 min at 4°C to remove platelets and cell debris. Then, plasma (2 ml) was resuspended in PBS (25 ml) and precleared by centrifugation at 20,000 rpm for 120 min at 4°C. The supernatants were passed through a 0.22-μm filter (Millipore). To isolate exosomes, the precleared supernatants of plasma were ultracentrifuged at 110,000 rpm for 120 min at 4°C, followed by washing of the exosome pellet with PBS at 110,000 rpm for 120 min at 4°C (Optima XPN-80 Ultracentrifuge, Beckman Coulter, SW32 rotor). The supernatant was discarded, and the exosome pellet was resuspended in PBS or lysed with Qiazol and stored at −80°C. Exosomes were characterized by dynamic light scattering.

### Dynamic Light Scattering

Exosomes were suspended in PBS at a concentration of 50 μg/ml, and analyses were performed with a Zetasizer Nano ZS (Malvern Instruments, Ltd.). Intensity, volume, and distribution data for each sample were collected on a continuous basis for 4 min in sets of 3.

### Western Blotting

Soluble exosome or cell lysates (5 μg) were resolved by SDS-PAGE (10–15%) and transferred to polyvinylidene fluoride membranes (Millipore). Blots were blocked for 3 h with 5% milk in Tris-buffered saline (TBS) with 0.1% Tween-20 and blotted overnight with the primary antibody directed to CD63 (#106228D, Invitrogen) in blocking solution. After three washes with TBS/0.1% Tween-20, filters were incubated for 1 h at room temperature with an HRP-conjugated secondary antibody before being revealed with an ECL substrate (Pierce Biotechnology).

### Small RNA Library Preparation, Sequencing, and Analysis

Total RNA was extracted with the miRNeasy kit (Qiagen) following the manufacturer’s protocol. Small RNA libraries (miRNA-seq) were prepared for sequencing using the Clontech SMARTer smRNA-Seq Kit from Illumina. Libraries were sequenced on an Illumina NextSeq instrument to obtain single-end 76-nt reads. RNA-seq reads from small RNA libraries were filtered and trimmed using Cutadapt according to Clontech recommendations (adapters and the first 3 nt were removed, and only reads 15 nt or longer were retained). Reads were mapped to the human genome (hg38) using Bowtie with a seed length of 19 with one mismatch, quantified for annotated miRNAs (miRBase). Using iDEP (integrated Differential Expression and Pathway analysis) software, miRNA reads were normalized; principal component analysis (PCA) was performed, and differential expression was assessed with DESeq2. Heat map of differentially expressed miRNAs was performed using the ClustVis tool.

### miRNA Target Prediction and Pathway Analysis

Ingenuity Pathway Analysis (IPA) software was used in order to assess diseases, biological processes, and pathway signaling associated with ARDS-related exosomal miRNAs.

Relative Quantification of miRNAs by Quantitative RT-PCR (qRT-PCR).

For quantification of miRNA expression, 50 ng RNA was reverse transcribed into cDNA using a qScript miRNA cDNA Synthesis Kit (Quanta Biosciences), and qRT-PCR was conducted in triplicate using Perfecta SYBR Green Super Mix (Quanta Biosciences). Thermal cycling was performed on an Applied Biosystems 7900 HT detection system (Applied Biosystems). Relative miRNA levels were normalized to three internal controls (miR-191-5p, miR-93-5p, and RNU-43) selected *via* small RNA sequencing data. The levels of these internal controls did not vary between ARDS cases and healthy controls in the validation cohort (Supplementary Figure S1).

### Statistical Analysis

Statistical analyses were performed using GraphPad Prism version 9. A *p*-value less than 0.05 was considered to be statistically significant. For the analysis of subject characteristics, the values are expressed as mean ± standard deviation. Statistical significance was determined using an unpaired *t*-test (continuous variables) or chi-square test (categorical variables). For comparison of miRNA expression in exosomes from ARDS patients vs. HSs, data were non-normally distributed and analyzed using the non-parametric two-tailed Mann–Whitney test. Individual data points are shown in all plots and represent data from independent samples. Receiver-operating characteristic (ROC) curves were plotted to investigate diagnostic value of altered miRNAs. The area under the curve (AUC) was calculated to evaluate the performance of these miRNAs in predicting ARDS.

## Results

### Identification of Exosomal miRNAs Differentially Expressed in the Plasma of ARDS Patients Compared With Healthy Subjects

To identify altered exosomal miRNAs in the ARDS context, we randomly divided 30 ARDS samples and 23 healthy subjects (HSs) into two cohorts: the discovery cohort (8 ARDS samples and 10 HSs) and the validation cohort (15 ARDS samples and 20 HSs) (Figure 1A). The patient characteristics are summarized in Table 1.

First, we identified altered exosomal miRNAs from the discovery cohort by small RNA sequencing (small RNA-seq). Briefly, plasma-derived exosomes were isolated using the standard ultracentrifugation protocol (as previously used by Njock et al. (2019)). The isolated vesicles had an average size distribution of 52 nm (±8.7) (Figure 1B) and 73 nm (±35) (Figure 1C) for HSs and ARDS samples, respectively, which is the typical size distribution of exosomes. Furthermore, these isolated vesicles present enrichment of the exosomal marker CD63, which confirms the purity of our plasma-derived exosome preparations (Figure 1D).

Then, the expression of exosomal miRNAs from ARDS- and HS-derived exosomes was assessed by small RNA-seq. After alignment, quantification, and normalization of reads from small RNA-seq data, we performed principal component analysis (PCA) which revealed that the repertoire of miRNAs from ARDS exosomes clustered distinctly from that of HS exosomes (Figure 1E). Differential expression analysis by DESeq2 allowed us to identify 17 miRNAs differentially expressed between ARDS samples and HSs (adjusted *p* < 0.1) (Figures 1F,G and Supplementary Table S1). We focused our attention on 12 exosomal miRNAs presenting the most pronounced alteration (adjusted *p* < 0.05): ten upregulated miRNAs (miR-142-5p, miR-122-5p, miR-223-3p, Let-7d-3p, miR-24-3p, miR-130a-3p, miR-98-3p, miR-221-3p, miR-193a-5p, and miR-1273a) and two downregulated miRNAs (Let-7a-5p and miR-486-5p) (Table 2).

**TABLE 2:** Differentially expressed exosomal miRNAs among HSs and ARDS patients in the discovery cohort.

MicroRNAs	Fold change	Adjusted *p*-values
**Upregulated miRNAs in ARDS**		
hsa-miR-142-5p	8.05	5.87E-06
hsa-miR-122-5p	19.00	1.03E-04
hsa-miR-223-3p	3.94	5.06E-04
hsa-Let-7d-3p	3.17	2.32E-03
hsa-miR-24-3p	4.77	2.32E-03
hsa-miR-130a-3p	5.67	4.13E-03
hsa-miR-98-3p	20.64	9.10E-03
hsa-miR-221-3p	9.78	0.013
hsa-miR-193a-5p	22.71	0.020
hsa-miR-1273a	15.34	0.022
**Downregulated miRNAs in ARDS**		
hsa-Let-7a-5p	−1.64	0.011
hsa-miR-486-5p	−3.29	0.022

RNA-seq analysis of exosomal miRNAs from the plasma of ARDS patients (*n* = 8) compared with HSs (*n* = 10).

### Biological Pathways Associated With ARDS-Related miRNAs

**FIGURE 2:**
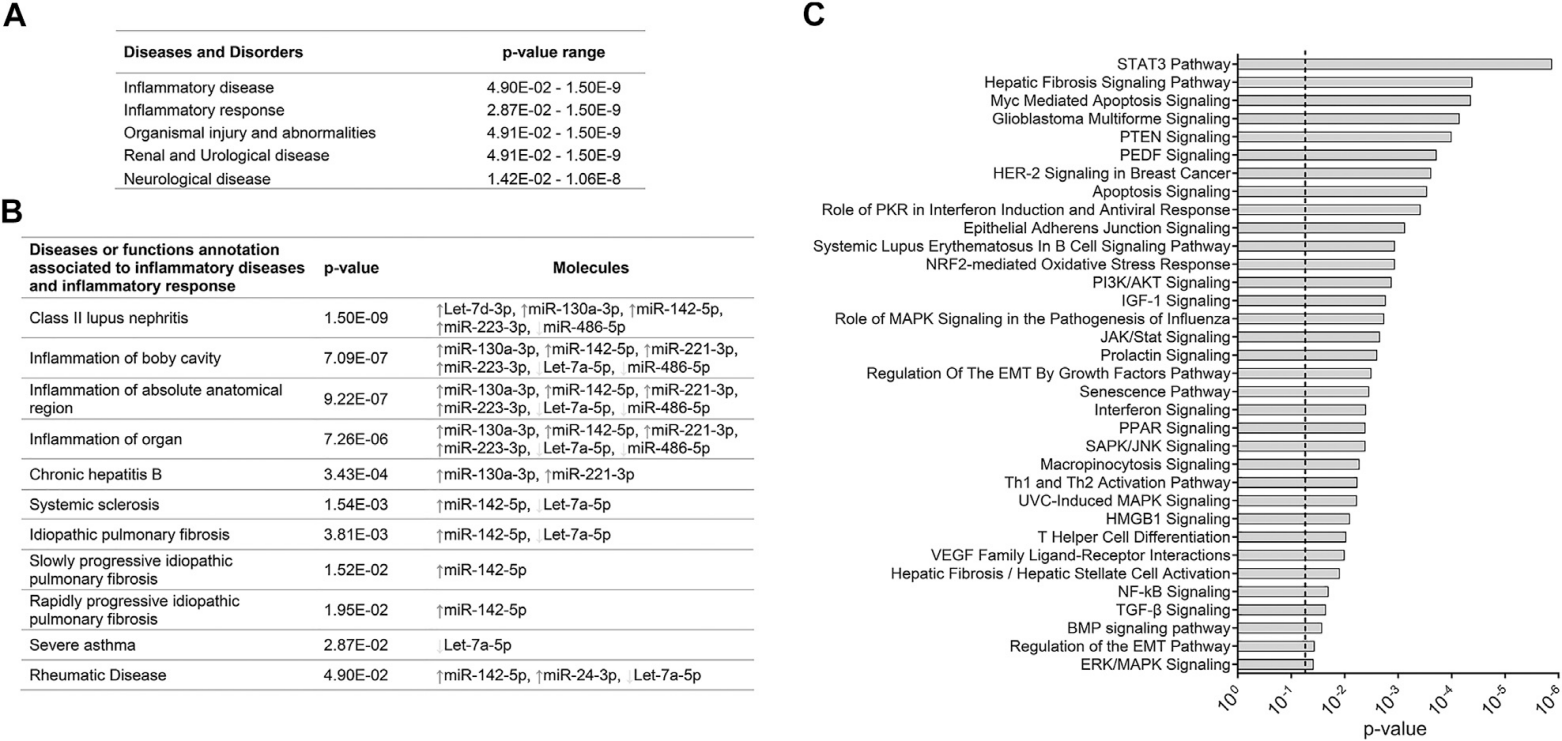
Canonical pathways associated with exosomal miRNAs altered in the plasma of ARDS patients. **(A)** Diseases and disorders associated with ARDS-exosomal miRNAs. **(B)** Disease or function annotation related to inflammatory response associated with ARDS-exosomal miRNAs. **(C)** Canonical pathways associated with ARDS-exosomal miRNAs. Seven ARDS-related miRNAs that showed robust changes (five upregulated miRNAs: miR-142-5p, miR-122-5p, miR-223-3p, Let-7d-3p, and miR-24-3p, and two downregulated miRNAs: Let-7a-5p and miR-486-5p) were subjected to the IPA tool.

In order to assess pathophysiological processes associated with ARDS-miRNAs, we submitted the list of 12 altered miRNAs (adjusted *p* < 0.05) to Ingenuity Pathway Analysis (IPA) software. According to IPA, ARDS-miRNAs are involved in the development of inflammatory diseases (*p* range 4.90E-02–1.50E-9), such as systemic sclerosis (*p* = 1.54E-03), idiopathic pulmonary fibrosis (*p* = 3.81E-03), and severe asthma (*p* = 2.87E-02) (Figures 2A,B). Six ARDS-miRNAs (four upregulated: miR-130a-3p, miR-142-5p, miR-221-3p, and miR-223-3p; two downregulated: Let-7a-5p and miR-486-5p) are associated with inflammation of the body cavity (*p* = 7.09E-07) and organ (*p* = 7.26E-06) (Figure 2B).

Furthermore, we investigated biological processes impacted by ARDS-miRNAs by submitted seven miRNAs presenting robust changes (five upregulated miRNAs: miR-142-5p, miR-122-5p, miR-223-3p, Let-7d-3p, and miR-24-3p, and two downregulated miRNAs: Let-7a-5p and miR-486-5p) to the IPA tool. Interestingly, several biological processes highlighted in this analysis are in agreement with ARDS pathophysiology, such as immune cell activation (Th1 and Th2 activation pathway, *p* = 5.88E-3; T helper cell differentiation, *p* = 9.33E-3; interferon signaling, *p* = 3.98E-3), fibrosis (hepatic fibrosis signaling pathway, *p* = 4.07E-5), and apoptosis/senescence (apoptosis signaling, *p* = 2.88E-4; senescence pathway, *p* = 3.46E-3) (Figure 2C). The signaling pathways that are likely impacted by ARDS-miRNAs are STAT3, PI3K/AKT, MAPK, and NF-kB pathways (*p* < 0.05) (Figure 2C).

### Validation of Exosomal miRNAs Related to ARDS in an Independent Cohort

Having identified altered miRNAs from the plasma of ARDS patients, we aimed to validate these candidates in an independent cohort (validation cohort) comprised of 15 ARDS patients and 20 healthy age- and sex-matched control subjects (Table 1) by quantitative real-time PCR. We used two miRNAs which were not differentially expressed in the discovery cohort, miR-191-5p (FC 1.09, adjusted *p* = 0.93) and miR-93-5p (FC 0.93, adjusted *p* = 0.93), as well as RNU-43 as endogenous controls to normalize miRNA expression levels. Several studies have demonstrated the high stability of these small non-coding RNAs in the plasma and recommended them as suitable endogenous normalizers for circulating miRNA quantification (Zheng et al., 2013; Barry et al., 2015; Korma et al., 2020). Having confirmed that the levels of miR-191-5p, miR-93-5p, and RNU-43 did not vary between ARDS cases and HSs in the validation cohort (Supplementary Figure S1), we used these small RNAs to normalize miRNA expression levels.

**FIGURE 3:**
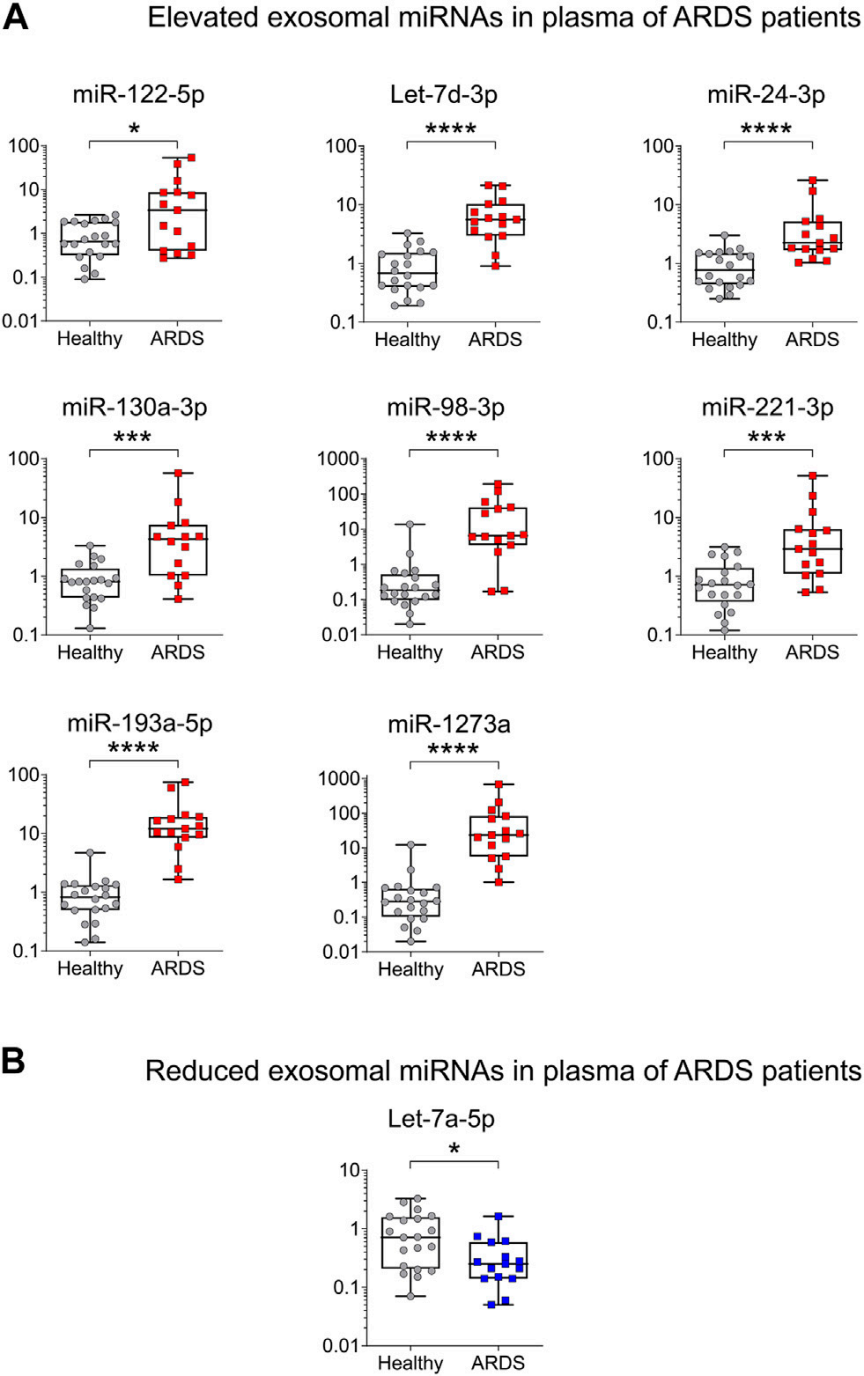
Validation of ARDS-related miRNAs in an independent cohort by qRT-PCR. **(A)** High levels of plasma exosomal miRNAs in ARDS. miR-122-5p Fold change (FC) = 5.17, Let-7d-3p FC = 8.22, miR-24-3p FC = 2.92, miR-130a-3p FC = 5.28, miR- 98-3p FC = 5.28, miR-221-3p FC = 4.10, miR-193a-5p FC = 14.79 and miR-1273a FC = 83.17. **(B)** Low levels of plasma exosomal miRNAs in ARDS. Let-7a-5p FC = −2.74. Data are non-normally distributed and analyzed using a non-parametric two-tailed Mann–Whitney test: *p < 0.05, **p < 0.01, ***p < 0.001, and ****p < 0.0001. HS (gray circles) *n* = 20 biologically independent samples; ARDS (red squares) *n* = 15 biologically independent samples.

Higher levels of miR-122-5p, Let-7d-3p, miR-24-3p, miR-130a-3p, miR-98-3p, miR-221-3p, miR-193a-5p, and miR-1273a were found in exosomes from ARDS blood, while Let-7a-5p presented a lower level as expected (Figures 3A,B), replicating the findings from the discovery cohort. Of the 12 miRNAs tested, three were not replicated. Indeed, the levels of miR-142-5p, miR-223-3p, and miR-486-5p presented no change between ARDS patients and HSs (Supplementary Figure S2).

### Diagnostic Potential of Plasma-Derived Exosomal miRNAs

**FIGURE 4:**
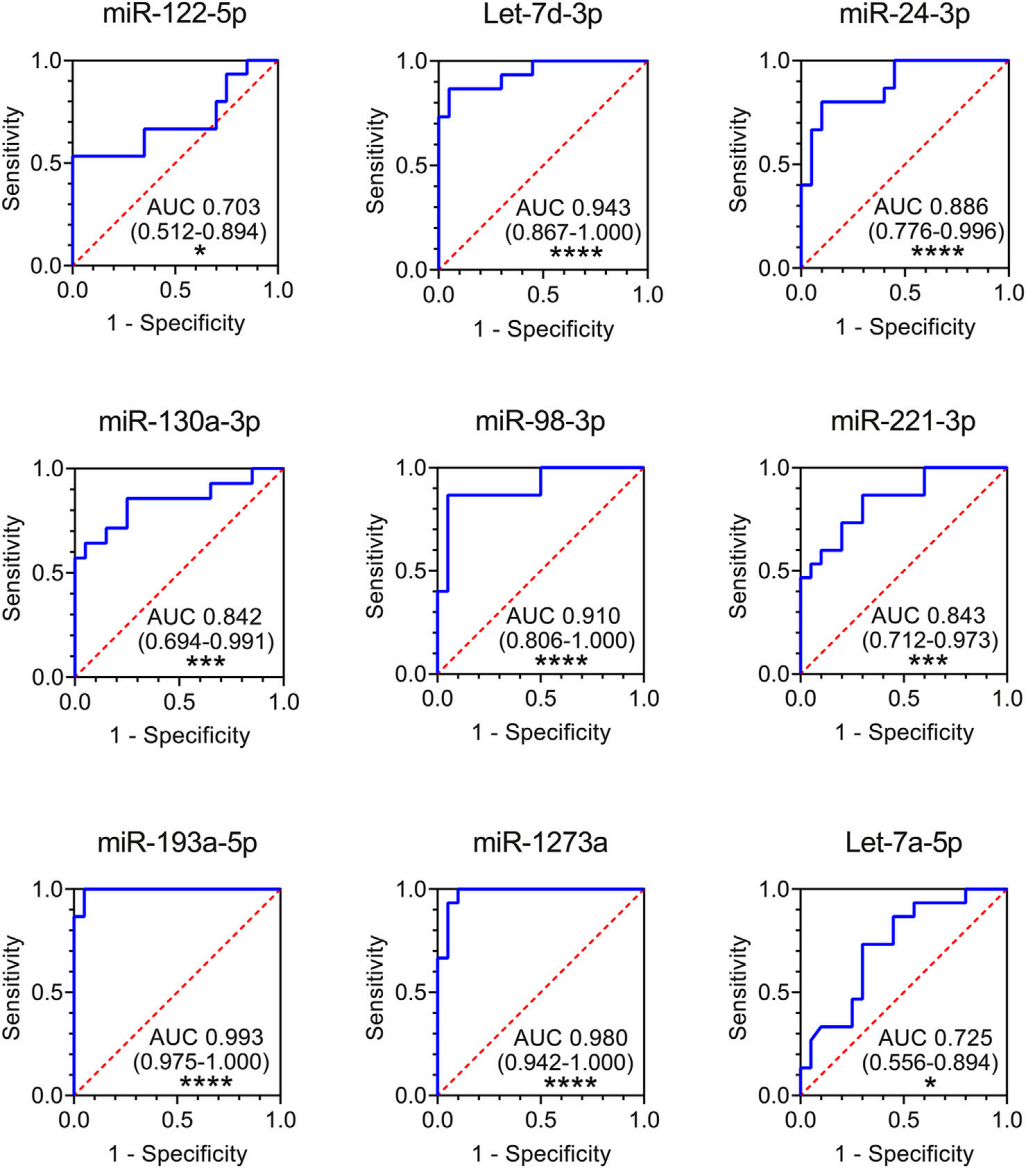
ROC curves for comparing the ability of exosomal miR-122-5p, Let-7d-3p, miR-24-3p, miR-130a-3p, miR-98-3p, miR-221-3p, miR-193a-5p, miR-1273a, and Let-7a-5p to discriminate ARDS patients from HSs. Area under the curves (AUCs) and 95% confidence interval (CI) of AUC are indicated. *p < 0.05, **p < 0.01, ***p < 0.001, and ****p < 0.0001. HS *n* = 20 biologically independent samples; ARDS *n* = 15 biologically independent samples (validation cohort).

Finally, to evaluate whether altered exosomal miRNAs could be used as potential diagnostic biomarkers for ARDS, we performed receiver-operating characteristic (ROC) curve analysis using the data from the validation cohort. The results showed that all the nine plasma exosomal miRNAs could serve as potential markers to discriminate ARDS patients from healthy controls, with the area under the curve (AUC) ranging from 0.703 (miR-122-5p) to 0.993 (miR-193a-5p) (Figure 4). Seven exosomal miRNAs presented high capability in distinguishing ARDS patients from HSs (AUC>0.8): miR-130a-3p (AUC 0.842), miR-221-3p (AUC 0.843), miR-24-3p (AUC 0.886), miR-98-3p (AUC 0.910), Let-7d-3p (AUC 0.943), miR-1273a (AUC 0.980), and miR-193a-5p (AUC 0.993) (Figure 4).

## Discussion

ARDS is a common cause of respiratory failure in critically ill patients, induced by acute inflammatory response in the lung. As the diagnosis and treatment of ARDS is complex, there is a need to develop novel biomarkers to identify early patients in order to deliver more appropriate treatment and new potential therapeutic targets. Here, for the first time, we characterized miRNA content of plasma exosomes from ARDS patients by small RNA-seq. We identified and validated the alteration of nine miRNAs in plasma exosomes from ARDS patients compared to HSs. ROC curve analysis showed that seven of them could discriminate ARDS patients from HSs with a high level of accuracy (AUC>0.8): miR-130a-3, miR-221-3p, miR-24-3p, miR-98-3p, Let-7d-3p, miR-1273a, and miR-193a-5p (Figure 4). These findings highlight exosomal miRNA dysregulation in the plasma of ARDS patients and provide candidate biomarkers for the diagnosis of ARDS disease.

In ARDS, only a few studies have investigated the identification of altered circulating miRNAs. Rahmel et al. (2018) have shown that circulating miR-122 is elevated in ARDS patients compared to controls and associated with mortality and acute liver injury. In another study, Xu et al. (2020) have shown that serum miR-92a expression is higher in patients with sepsis-induced ARDS, when compared to patients with sepsis only. Recently, Zhu et al. (2017a) profiled miRNA expression in blood preparation from ARDS patients and critically ill at-risk controls and identified three altered miRNAs (miR-424, miR-92a, and miR-181a). To date, no study has characterized miRNA content from exosomes of ARDS patients. There is just one study where the levels of selected miRNAs from blood exosomes of ARDS patients were assessed. Indeed, Wu et al. (2019) investigated the expression of ten ARDS-relevant miRNAs (obtained by literature search) from the serum of patients with/without ARDS and identified a subset of four altered miRNAs (miR-146a, miR-27a, miR-126, and miR-155).

To our knowledge, our study is the first to profile miRNAs from plasma exosomes of ARDS patients by small RNA-seq in order to identify the ones that are the most altered. We identified several miRNAs that have already been described as potential biomarkers or involved in the development of ARDS (miR-122-5p (Rahmel et al., 2018), miR-221-3p (Zheng et al., 2018), miR-486-5p (Luo et al., 2020), miR-24-3p, and Let-7a-5p (Huang et al., 2014)), but we have also some miRNAs that have not yet been associated with the disease (miR-130a-3p, miR-193a-5p, or miR-1273a) (Figures 1, 3). The functional study of these miRNAs could open new avenues for the development of therapeutics.

IPA revealed that most of ARDS-related miRNAs are associated with different processes implicated in the pathobiology of the disease, mostly inflammation of the body cavity (*p* = 7.09E-07) and organ (*p* = 7.26E-06) (Figure 2B). Interestingly, there are few overlapping miRNAs in ARDS and IPF. Indeed, two altered miRNAs have been associated with IPF: miR-142-5p and Let-7a-5p (Figure 2B). Yang et al. (2015) have shown that Let-7a-5p was significantly decreased in the serum of IPF patients compared to HSs. Another study revealed that miR-130a-3p and miR-142-5p, which were dysregulated in macrophages from tissue samples of IPF patients, were able to sustain the profibrogenic effect of macrophages and therefore might be involved in the development of pulmonary fibrosis (Su et al., 2015).

Recent studies confirmed involvement of these miRNAs in the induction of ARDS-related processes. miR-122, mainly from hepatic origin, plays a role in the regulation of the hepatocyte by controlling cellular proliferation, differentiation, and apoptosis (Jopling, 2012). Furthermore, it has been shown that miR-122 is a specific serum biomarker for hepatocyte damage (Verma et al., 2016). Moreover, the association of APACHE II score and elevated serum miR-122 expression level is associated with disease severity and prognosis of ARDS patients (Hao et al., 2019). The functional study of miR-122-5p in the LPS-induced acute lung injury (ALI) mouse model revealed that a high level of miR-122-5p acted as a pathologic factor that exaggerated lung injury, inflammation, and oxidative stress and promoted ALI progression (Lu et al., 2020). Thus, miR-122-5p inhibition may be a potential treatment strategy for ALI caused by Gram-negative bacterial infection. To conclude, this upregulated biomarker is therefore above all an element of severity in our cohort, reflecting states of multiorgan failure.

miRNAs have been reported to be involved in the inflammatory response in many ways. With regard to miR-221-3p, its upregulation has been shown to play a role in the inflammatory response and the development of ALI in an LPS-induced mice model (Wang et al., 2019). Furthermore, increasing evidence suggests that extracellular vesicle–enriched miR-221-3p is the key factor in the pathogenesis of ARDS. Indeed, these vesicles actively delivered miR-221-3p into alveolar macrophages, subsequently promoting inflammasome activation, neutrophil recruitment, and M1-macrophage polarization (Lee et al., 2016, 2017; Zhu et al., 2017b). Another study reported that miR-221-3p induces the shift of M2-macrophages to a pro-inflammatory phenotype (Quero et al., 2020). The inhibition of miR-221-3p could therefore have a probable protective effect on ARDS progression.

miR-24-3p has also been implicated in ARDS. Unlike the work carried out on ARDS models in rats (Huang et al., 2014), we note that miR-24-3p is upregulated in our cohort. Moreover, it has been shown that overexpression of miR-24-3p significantly reduced LPS-induced lung damage and decreased the release of pro-inflammatory cytokines in the rat model (Lin and Yang, 2019). This complexity between pro-inflammatory and anti-inflammatory balance must still be understood *in vivo*.

It has been clearly demonstrated that the upregulation of miR-130a-3p plays a role in the differentiation of cardiac myofibroblasts by targeting the PPAR gamma pathway (Li et al., 2017). We can therefore imagine that this pathway is also involved in balance fibrosis/anti-fibrosis occurring in ARDS disease.

The downregulation of Let-7a-5p has been observed in a previous study in the rat model of ARDS (Huang et al., 2014). Furthermore, Let-7a-5p has been reported to suppress cell growth in multiple cancer types (Zhao et al., 2018); however, the pathophysiological mechanism is not yet known. One study suggests that Let-7d-3p exerts protective effects on cardiac cells subjected to hypoxia stress by inhibiting apoptosis (Wong et al., 2019). Future work has to be carried out in order to specify the role of Let-7a-5p and Let-7d-3p in ARDS development.

miR-193a-5p was found to be upregulated in a mouse model of ALI induced by inhalation of staphylococcal enterotoxin B. Pathway analysis revealed that miR-193a-5p is involved in ALI progression by targeting several molecules of transforming growth factor-beta (TGF-β) signaling (TGF-β2, TGFβ-R3) and apoptotic signaling pathways (Alghetaa et al., 2018).

To decipher molecular mechanisms that could contribute to the disease, we investigated signaling pathways that may be impacted by ARDS-miRNAs. The main pathways targeted are STAT3, PI3K/AKT, MAPK, and NF-kB pathways (*p* < 0.05) (Figure 2). Interestingly, several studies have shown that these signaling pathways are involved in the development of ARDS. Indeed, the impact of NF-kB on ARDS progression is well studied (Meduri et al., 2005; Hariharan et al., 2020), as well as PI3K/AKT (Y et al., 2020) and MAPK (Newton and Holden, 2006). Recently, Zhang et al. (2019) found that STAT3 may be the key regulatory gene in the underlying dysfunction of sepsis-induced ARDS. The mRNA targets associated with ARDS-miRNAs are potential candidates for development of new therapeutic strategies.

In conclusion, we identified potential biomarkers for the diagnosis of ARDS from plasma-derived exosomes. From the nine altered miRNAs identified, seven present a good capacity to discriminate ARDS patients from HSs. These miRNAs are involved in different biological processes associated with the development of the disease such as inflammation or fibrosis. The biomarker status of these exosomal miRNAs needs to be validated in large independent cohorts. In addition, their potential value on early detection of ARDS needs to be evaluated in future studies. This study provides the basis to improve ARDS diagnosis and open avenues to develop new therapeutic strategies.

## Data Availability

The datasets presented in this study can be found in online repositories. The name of the repository and accession number can be found in the ArrayExpress database under accession number E-MTAB-9991.
