# Supplementary material for: A Blood Exosomal miRNA Signature in Acute Respiratory Distress Syndrome
Source: Front Mol Biosci. 2021 Jul 15;8:640042. doi: 10.3389/fmolb.2021.640042 (PMC8319727; doi:10.3389/fmolb.2021.640042)
Supplement: Supplementary file 2 [file DataSheet1.PDF]

Supplementary Material

A Blood Exosomal MiRNA Signature in Acute Respiratory Distress Syndrome

Gilles Parzibut[1], Monique Henket[2], Catherine Moermans[2], Ingrid Struman[3], Edouard Louis[4,6], Michel Malaise[5,6], Renaud Louis[2,6], Benoît Misset[1], Makon-Sébastien Njock[2,4,5,6]† and Julien Guiot[1,2,6]†*

†These authors have contributed equally to this work and share last authorship

Affiliations :

[1]Department of Intensive Care, University Hospital of Liège, Liège, Belgium. [2]Laboratory of Pneumology, GIGA Research Center, University of Liège, University Hospital of Liège, Liège, Belgium. [3]Laboratory of Molecular Angiogenesis, GIGA Research Center, University of Liège, Liège, Belgium. [4]Laboratory of Gastroenterology, GIGA Research Center, University of Liège, University Hospital of Liège, Liège, Belgium. [5]Laboratory of Rheumatology, GIGA Research Center, University of Liège, University Hospital of Liège, Liège, Belgium. [6]Fibropole Research Group, University Hospital of Liège, Liège, Belgium

*** Correspondence:**

Dr Makon-Sébastien Njock
ms.njock@chuliege.be
Laboratory of Pneumology, GIGA Research
University of Liege
University Hospital of Liege
Liege, Belgium

SUPPLEMENTARY DATA

Supplementary file 1. DESeq2 analysis of full list of miRNAs identified. Comparison between ARDS (n=8) vs HS (n=10). Log2 fold changes and adjusted p-values are included for each miRNA.

Supplementary file 2. Full list of canonical pathways associated to exosomal miRNAs altered in the plasma of ARDS patients. Lists of molecules associated to altered miRNAs are included for each canonical pathway.

SUPPLEMENTARY FIGURES

Supplementary Figure 1. Raw Ct values in healthy and ARDS groups for endogenous control small RNAs. Raw Ct values were plotted for the three endogenous controls (miR-191-5p, miR-93-5p and RNU-43), and are shown with mean and standard deviation in both healthy and ARDS groups.

Supplementary Figure 2. Relative expression of miR-142-5p, miR-223-3p and miR-486-5p for healthy and ARDS groups.

SUPPLEMENTARY TABLES

Supplementary Table 1. Differentially expressed exosomal miRNAs among HS and ARDS patients in the discovery cohort (adjusted $p < 0.1$).

Supplementary Table 2. Primers used for qRT-PCR.

SUPPLEMENTARY FIGURES

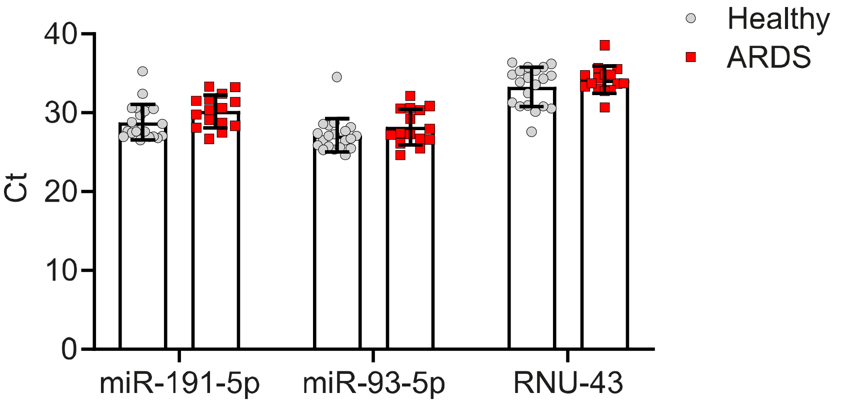

Supplementary Figure S1. Raw Ct values in healthy and ARDS groups for endogenous control small RNAs. Raw Ct values were plotted for the three endogenous controls (miR-191-5p, miR-93-5p and RNU-43), and are shown with mean and standard deviation in both healthy and ARDS groups.

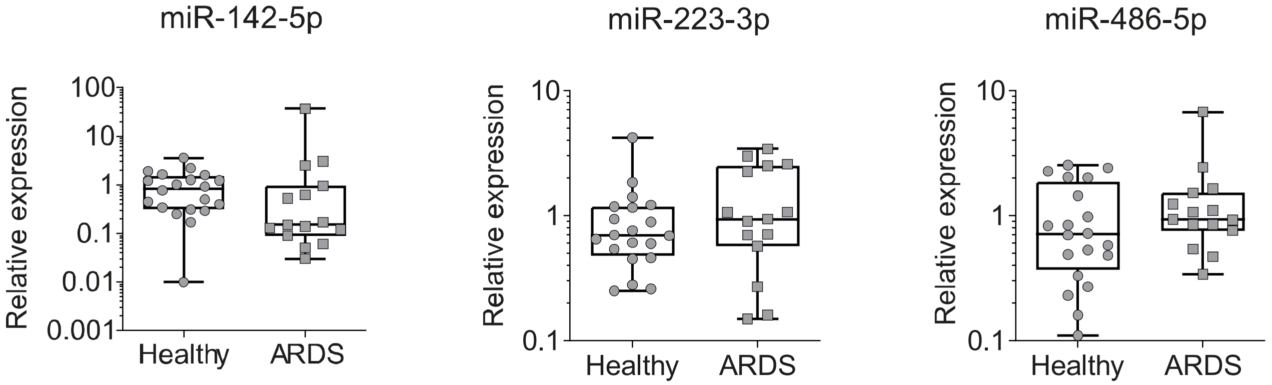

Supplementary Figure S2. Relative expression of miR-142-5p, miR-223-3p and miR-486-5p for healthy and ARDS groups.

SUPPLEMENTARY TABLES

Supplementary Table 1. Differentially expressed exosomal miRNAs among HS and ARDS patients in the discovery cohort (adjusted $p < 0.1$).

MicroRNAs	Fold change	Adjusted p-values
**Upregulated miRNAs in ARDS**		
hsa-miR-142-5p	8.05	5.87E-06
hsa-miR-122-5p	19.00	1.03E-04
hsa-miR-223-3p	3.94	5.06E-04
hsa-Let-7d-3p	3.17	2.32E-03
hsa-miR-24-3p	4.77	2.32E-03
hsa-miR-130a-3p	5.67	4.13E-03
hsa-miR-98-3p	20.64	9.10E-03
hsa-miR-221-3p	9.78	0.013
hsa-miR-193a-5p	22.71	0.020
hsa-miR-1273a	15.34	0.022
hsa-miR-146A-5p	4.94	0.056
hsa-Let-7b-3p	2.72	0.056
hsa-miR-335-5p	8.02	0.062
hsa-miR-483-5p	11.40	0.084
hsa-miR-28-3p	5.88	0.096
**Downregulated miRNAs in ARDS**		
hsa-Let-7a-5p	-1.64	0.011
hsa-miR-486-5p	-3.29	0.022

Supplementary Table 2. Primers used for qRT-PCR

microRNA	Forward Primer (5'-->3')	Reverse Primer (5'-->3')
hsa-miR-142-5p	CATAAAGTAGAAAGCACTACT	Universal primer
hsa-miR-122-5p	TGGAGTGTGACAATGGTGTTTG	Universal primer
hsa-miR-223-3p	TGTCAGTTTGTCAAATACCCCA	Universal primer
hsa-Let-7d-3p	CTATACGACCTGCTGCCTTTCT	Universal primer
hsa-miR-24-3p	TGGCTCAGTTCAGCAGGAACA	Universal primer
hsa-miR-130a-3p	CAGTGCAATGTTAAAAGGGCATA	Universal primer
hsa-miR-98-3p	CTATACAACTTACTACTTTCCC	Universal primer
hsa-Let-7a-5p	TGAGGTAGTAGGTTGTATAGTT	Universal primer
hsa-miR-221-3p	AGCTACATTGTCTGCTGGGTTTC	Universal primer
hsa-miR-193a-5p	TGGGTCTTTGCGGGCGAGATGA	Universal primer
hsa-miR-1273a	GGGCGACAAAGCAAGACTCTTTCTT	Universal primer
hsa-miR-486-5p	TCCTGTACTGAGCTGCCCCGAG	Universal primer
hsa-miR-191-5p	CAACGGAATCCCAAAAGCAGCTG	Universal primer
hsa-miR-93-5P	CAAAGTGCTGTTCGTGCAGGTAG	Universal primer
hsa-RNU43	CTTATTGACGGGCGGACAGAAAC	Universal primer
